# Probiotic Therapy With VSL#3^®^ in Patients With NAFLD: A Randomized Clinical Trial

**DOI:** 10.3389/fnut.2022.846873

**Published:** 2022-05-24

**Authors:** Giuseppe Derosa, Luigina Guasti, Angela D’Angelo, Chiara Martinotti, Maria Chiara Valentino, Sergio Di Matteo, Giacomo M. Bruno, Andrea M. Maresca, Giovanni V. Gaudio, Pamela Maffioli

**Affiliations:** ^1^Center of Diabetes and Metabolic Diseases, Department of Internal Medicine and Therapeutics, University of Pavia, Pavia, Italy; ^2^Italian Nutraceutical Society (SINut), Bologna, Italy; ^3^Geriatric Division, ASST dei Sette Laghi, University of Insubria, Varese, Italy; ^4^S.A.V.E. Studi Analisi Valutazioni Economiche Research Center, Milan, Italy; ^5^Department of Management Information and Production Engineering, University of Bergamo, Bergamo, Italy; ^6^Medical Division, ASST dei Sette Laghi, University of Insubria, Varese, Italy; ^7^Internal Medicine Division, Ospedale Angelo Bellini, Varese, Italy

**Keywords:** NAFLD, VSL#3^®^, probiotics, steatosis, *Lactobacilli*

## Abstract

**Aim:**

To evaluate if VSL#3^®^ [a high-concentration multi-strain probiotic mix containing one strain of *Streptococcus thermophilus* BT01, three strains of *Bifidobacteria* (*B. breve* BB02; *B. animalis* subspecies [subsp.] *lactis* BL03, previously identified as *B. longum* BL03; and *B. animalis* subsp. *lactis* BI04, previously identified as *B. infantis* BI04), and four strains of *Lactobacilli* (*L. acidophilus* BA05, *L. plantarum* BP06, *L. paracasei* BP07, and *L. helveticus* BD08, previously identified as *L. delbrueckii* subsp. *bulgaricus* BD08)] therapy could improve hepatic parameters.

**Methods:**

We enrolled 60 Caucasian patients aged ≥ 18 years of either sex with the diagnosis of non-alcoholic fatty liver disease (NAFLD), according to practice guidance, in a double-blind, placebo-controlled study. Patients were randomized to take placebo or VSL#3^®^, 2 sachets/day in the morning for 3 months. VSL#3^®^ and placebo were self-administered.

**Results:**

We did not observe any change in body mass index (BMI), circumferences, fasting plasma glucose (FPG), total cholesterol (TC), low-density lipoprotein-cholesterol (LDL-C), high-density lipoprotein-cholesterol (HDL-C), and adiponectin (ADN) with neither treatment. A statistically significant triglycerides (Tg) decrease (*p* < 0.05 vs. baseline, and *p* < 0.05 vs. placebo, respectively) and high-sensitivity C-reactive protein (Hs-CRP) decrease (*p* < 0.05 vs. baseline) was observed in the group of patients being treated with VSL#3^®^ compared with placebo. Transaminases and gamma-glutamyltransferase (γ-GT) were significantly reduced in VSL#3^®^ group (*p* < 0.05 vs. baseline and placebo, respectively) compared with the placebo group. Aspartate aminotransferase (AST)/alanine aminotransferase (ALT) ratio and hepatic steatosis index (HSI) were significantly lower than the VSL#3^®^ group (*p* < 0.05 vs. baseline and placebo, respectively) compared with the placebo group. All patients reported an improvement or the disappearance of hepatic steatosis.

**Conclusion:**

Probiotic therapy with VSL#3^®^ ameliorates hepatic parameters and echography grading, while reducing Tg and the inflammatory status, without any difference between men and women.

## Introduction

Non-alcoholic fatty liver disease (NAFLD) is a metabolic disorder characterized by hepatic fat accumulation in the absence of significant alcohol consumption (1).

NAFLD is currently the main cause of chronic liver disease in developed countries, and the number of patients with NAFLD is growing worldwide (2). NAFLD is a consequence of triglycerides (Tg) accumulation in the hepatocytes and is considered as the hepatic manifestation of obesity and metabolic syndrome (3). Until a few years ago, NAFLD was considered a benign condition, but now it is known that NAFLD can progress to non-alcoholic steatohepatitis (NASH), where inflammation and hepatocellular damage are associated with steatosis. NASH is associated with fibrogenesis and can develop into cirrhosis and hepatocellular carcinoma. Almost one-fourth of Italians aged between 18 and 65 years are affected by NAFLD, overall, the estimated prevalence of NAFLD in the general population is approximately 20–40%, with a higher prevalence in obese and diabetic patients (4). The quick progression of this condition is linked to the changes in a quantity of environmental factors interacting with genetic and epigenetic factors. Weight loss is the only proven effective strategy for the treatments of NAFLD (5), even if several therapeutic approaches have been proposed.

In Italy, an analysis aimed to evaluate the use of health resources and the costs associated with patients with NAFLD/NASH and advanced liver disease was conducted. The group included NAFLD/NASH patients with compensated cirrhosis (CC), decompensated cirrhosis (DCC), liver transplant (LT), or hepatocellular carcinoma (HCC). Researchers examined data from nearly 10,000 Italian patients with NAFLD/NASH who were hospitalized between 2011 and 2017 and identified 131 individuals (1.3%) with CC, 303 (3.1%) with DCC, 11 (0.1%) with LT, and 79 (0.8%) with HCC. NAFLD/NASH patients with advanced liver disease were hospitalized on average 4.2–4.4 times per year, compared with 2.9 times in those without advanced liver disease (*p* < 0.05). The average annual total healthcare costs associated with patients hospitalized with NAFLD/NASH were at least 86% higher in subjects with advanced liver disease than in those without, mainly due to higher hospital costs: € 10,576 for patients with NAFLD/NASH without advanced liver disease, € 19,681 for those with CC, € 19,808 for those with DCC, € 65,137 with LT, and € 26,220 with HCC (total annual costs 2017).

A similar trend was observed after adjusting costs for patient characteristics and comorbidities, such as type 2 diabetes and cardiovascular disease, suggesting that liver-related complications accounted for at least 50% of total health costs among patients with advanced liver disease: € 2,418 for those without advanced liver disease, compared with € 9.318 for those with CC, € 9.717 for DCC, € 55.677 for LT, and € 16.185 for HCC (*p* < 0.01 for CC, DCC, and HCC; *p* = 0.08 per LT).

The annual costs associated with NAFLD/NASH patients with advanced liver disease are extremely high and increase with the progression of liver disease, highlighting the need for effective interventions to prevent progression.

Current evidence suggests some nutraceuticals for the treatment of patients with NAFLD, such as vitamin D, vitamin E, carnitine, polyunsaturated fatty acids (PUFAs), silymarin, resveratrol, anthocyanins, and betaine (6). The role of the gut microbiota is becoming increasingly important as interface between environmental changes and host biology (7). Probiotics are defined as live microorganisms that, when administered in adequate amounts, confer a health benefit on the host (8). There is an anatomical link between the intestine and liver *via* the hepatic portal system. Based on the connection between the intestine and the liver, also termed as the gut-liver axis, gut microbiota and their metabolic products may influence liver pathology (9).

In literature, a previous proof-of-concept study conducted on a little number of patients was undertaken showing that VSL#3^®^ did not significantly improve the markers of cardiovascular risk and liver injury in patients with NAFLD after 10 weeks of treatment. However, the study supports an association between endothelial dysfunction and inflammation in patients with NAFLD (10). On this basis, we projected a larger and longer study aimed to evaluate if a supplementation with VSL#3^®^ [a high-concentration multi-strain probiotic mix containing one strain of *Streptococcus thermophilus* BT01, three strains of *Bifidobacteria* (*B. breve* BB02; *B. animalis* subspecies [subsp.] *lactis* BL03, previously identified as *B. longum* BL03; and *B. animalis* subsp. *lactis* BI04, previously identified as *B. infantis* BI04), and four strains of *Lactobacilli* (*L. acidophilus* BA05, *L. plantarum* BP06, *L. paracasei* BP07, and *L. helveticus* BD08, previously identified as *L. delbrueckii* subsp. *bulgaricus* BD08)] could improve hepatic parameters.

## Materials and Methods

### Study Design

This 3-months, double-blind, randomized, placebo-controlled study was conducted at the University of Insubria, Geriatric Division, ASST dei Sette Laghi, Varese, Italy, Ospedale Angelo Bellini, Somma Lombardo, Italy, and at the Department of Internal Medicine and Therapeutics, University of Pavia, Italy, among patients attending the Diabetes and Metabolic Diseases Center and the Center for Prevention, Surveillance, Diagnosis, and Treatment of Rare Diseases.

The study protocol was approved by the ethics committee of Insubria (Protocol n. 105/2020) and was conducted in accordance with the 1994 Declaration of Helsinki, and its amendments and the Code of Good Clinical Practice. All patients provided written informed consent to participate in this study after a full explanation of the study was given.

### Materials and Methods

From 7 July 2021 to 23 December 2021, we enrolled 60 Caucasian patients aged ≥ 18 years of either sex, with the diagnosis of NAFLD, according to practice guidance (11) and in particular with these characteristics:

-transaminases [aspartate aminotransferase (AST) and alanine aminotransferase (ALT)] increase,

-ratio inversion AST/ALT,

-absence of virus markers of hepatitis, and

-absence of alcohol consumption or consumption less than 20 g/day in women and 30 g/day in men.

For further and additional confirmation of the diagnosis of NAFLD, the hepatic steatosis index (HSI) has been calculated using the formula:

8 × (AST/ALT ratio) + BMI (+ 2 if woman; + 2 if diabetic)

The result of the formula could be < 30.0 or > 36.0 to further classify these patients as having NAFLD (12).

An additional criterion was hepatomegaly and/or the presence of hepatic echography with a framework of NAFLD, such as the hyper-reflective (bright) surface of the liver.

Suitable patients, identified from a review of case notes and/or computerized clinic registers, were contacted by the investigators in person or by telephone.

Patients were excluded if they have chronic liver disease; impaired renal function (defined as serum creatinine level higher than the upper limits of normal (ULN) for age and sex); endocrine disorders, or gastrointestinal disorders; current or previous evidence of ischemic heart disease, heart failure, or stroke; malignancy; and significant neurological or psychiatric disturbances, such as alcohol or drug abuse. Excluded medications (within the previous 3 months) were anorectic agents, laxatives, β-agonists (other than inhalers), diuretics, cyproheptadine, anti-depressants, anti-serotoninergics, phenothiazines, barbiturates, oral corticosteroids, and anti-psychotics. In addition, women who were pregnant or breastfeeding or of childbearing potential and not taking adequate contraceptive precautions were excluded.

### Diet and Physical Activity

At baseline, all patients were already following an adequate diet. The controlled-energy diet (∼600 kcal daily deficit) was based on NCEP-ATP III recommendations (13), that contained 50% of calories from carbohydrates, 30% from fat (< 7% saturated, up to 10% polyunsaturated, and up to 20% monounsaturated), and 20% from proteins, with a maximum cholesterol content of 300 mg/day, and 35 g/day of fiber. Standard diet advice was given by a dietitian and/or specialist physician. Additionally, individuals were encouraged to maintain their usual physical activity.

### Treatment

Patients were randomized to take placebo or VSL#3^®^, 2 sachets/day in the morning for 3 months. VSL#3^®^ and placebo were self-administered. Both VSL#3^®^ and placebo were supplied as identical, coded boxes to ensure the blind status of the study.

Randomization was done using a drawing of envelopes containing randomization codes prepared by a statistician. Medication compliance was assessed by counting the number of sachets returned at the time of specified clinic visits. Throughout the study, we instructed patients to take their first dose of new medication on the day after they were given the study medication. At the same time, all unused medication were retrieved for inventory. All medications were provided free of charge.

### Assessments

Before starting the study, all patients underwent an initial screening assessment that include a medical history, physical examination, vital signs (blood pressure and heart rate), a 12-lead electrocardiogram (ECG), measurements of height and body weight, calculation of body mass index (BMI), abdominal circumference (Abd. Cir.), waist circumference (Waist Cir.), and hip circumference (Hip Cir.), total cholesterol (TC), low-density lipoprotein-cholesterol (LDL-C), high-density lipoprotein-cholesterol (HDL-C), Tg, transaminases [AST and ALT], gamma-glutamyltransferase (γ-GT), high-sensitivity C-reactive protein (Hs-CRP), and adiponectin (ADN).

All parameters were assessed at baseline, and after 3 months since the start of study.

All parameters were determined in fasting state, after a 12-h overnight fast, in the plasma. Venous blood samples were taken for all patients between 8 and 9 a.m. and were drawn from an antecubital vein with a 19-gauge needle without venous stasis.

We used plasma obtained by the addition of Na_2_-EDTA, 1 mg/ml, and centrifuged at 3,000 *g* for 15 min at 4°C. Immediately after centrifugation, the plasma samples were frozen and stored at −80°C for no more than 3 months. All measurements were performed in a central laboratory.

The BMI was calculated by the investigators as weight in kilograms divided by the square of height in meters. Plasma glucose was assayed using a glucose-oxidase method (GOD/PAP, Roche Diagnostics, Mannheim, Germany) with intra- and inter-assay coefficients of variation (CsV) < 2% (14).

Total cholesterol and Tg levels were determined using fully enzymatic techniques (15, 16) on a clinical chemistry analyzer (HITACHI 737; Hitachi, Tokyo, Japan); intra- and inter-assay coefficient of variation (CsV) were 1.0 and 2.1 for TC measurement, and 0.9 and 2.4 for Tg measurement, respectively. HDL-C level was measured after the precipitation of plasma apo B-containing lipoproteins with phosphotungstic acid (17) intra- and inter-assay CsV were 1.0 and 1.9, respectively; LDL-C level was calculated by the Friedewald formula (18).

Transaminases and γ-GT were evaluated in a central laboratory according to standard methods. Hs-CRP was measured with the use of latex-enhanced immunonephelometric assays on a BN II analyzer (Dade Behring, Newark, DE, United States). The intra- and interassay CsV were 5.7 and 1.3%, respectively (19). Adiponectin level was determined using the ELISA kits (B-bridge International, Sunnyvale, CA, United States). Intraassay CsV were 3.6% for low- and 3.3% for high-control samples, whereas inter-assay CsV were 3.2% for low- and 7.3% for high-control samples, respectively (20).

### Hepatic Echography

Each patient underwent abdominal ultrasonography using a 3.0 MHz curved array transducer and a standard Acuson Sequoia 512 system (Acuson, Mountain View, CA, United States). Grading of the severity of fatty liver disease in patients with NAFLD was determined as described previously (21, 22). Level 0 was defined as a normal hepatic echo pattern, level 1 as a slight increase in echo pattern with the normal visualization of vessels and diaphragm, level 2 as a moderate increase in echogenicity with the reduced visibility of portal veins and diaphragm, and level 3 as a pronounced increase in hepatic echo pattern with the poor visibility of intrahepatic vessels and posterior right lobe of the liver.

### Safety Measurements

Treatment tolerability was assessed at each study visit using an accurate interview of patients by investigators, and the comparisons of clinical and laboratory values with baseline levels. Safety monitoring included physical examination, vital sign assessment, weight, adverse events, and laboratory tests. All adverse events were recorded.

### Statistical Analysis

We evaluated sample size using a sample size calculator made available by Epicentro, Istituto Superiore di Sanità (Italy). A difference of at least 10% compared with the baseline was considered clinically significant; considering an alpha error of 0.05, a confidence level of 95%, and a confidence interval (*CI*) of 12.65, the actual sample size is adequate to obtain a power higher than 0.80 for all variables.

Patients were included in the tolerability analysis if they received ≥ 1 dose of trial medication after randomization and underwent a subsequent tolerability observation. Continuous variables were tested using a two-way repeated measures analysis of variance (ANOVA). Intervention effects were adjusted for additional potential confounders using the analysis of covariance. In addition, ANOVA was used to assess the significance within and between groups. A statistical analysis of data was performed using the Statistical Package for Social Sciences software version 14.0 (SPSS Inc., Chicago, IL, United States). Data were presented as mean (SD). For all statistical analyses, the values of *p* < 0.05 were considered statistically significant (23).

## Results

### Study Sample

A total of 60 patients affected by NAFLD were enrolled in the trial. Of these, 30 were randomized to VSL#3^®^ supplementation and 30 to placebo. All the patients completed the study.

### Anthropometric Parameters

We did not observe any change in BMIs or circumferences in both the VSL#3^®^ supplementation or placebo treatment (Table 1).

**TABLE 1:** VSL#3^®^ treatment at baseline and after 3 months.

	Placebo				VSL#3			
	Baseline		3 months		Baseline		3 months	
Total number	30		30		30		30	
Sex	Males	Females	Males	Females	Males	Females	Males	Females
Number	13	17	13	17	15	15	15	15
Age (years)	55.8 ± 7.7	57.5 ± 7.9	-	-	55.7 ± 6.7	55.9 ± 7.5	-	-
Smoking status (M/F)	4	5	4	5	6	5	6	5
Height (m)	1.69 ± 0.07	1.67 ± 0.04	-	-	1.70 ± 0.07	1.68 ± 0.05	-	-
Weight (Kg)	75.3 ± 8.1	77.2 ± 8.7	74.6 ± 8.0	76.3 ± 8.2	75.1 ± 8.4	77.4 ± 8.6	74.5 ± 7.9	76.9 ± 8.1
BMI (Kg/m^2^)	26.4 ± 3.1	27.7 ± 3.7	26.1 ± 3.0	27.4 ± 3.5	26.0 ± 2.9	27.4 ± 3.5	25.8 ± 2.8	27.2 ± 3.4
Abd. Cir. (cm)	94.4 ± 3.0	94.8 ± 3.4	94.1 ± 2.9	94.5 ± 3.3	95.0 ± 3.6	95.4 ± 3.7	94.1 ± 2.9	94.6 ± 3.4
Waist Cir. (cm)	89.1 ± 2.6	89.3 ± 2.9	88.2 ± 2.4	88.7 ± 2.5	89.5 ± 3.0	89.7 ± 3.1	88.1 ± 2.3	88.8 ± 2.8
Hip Cir. (cm)	100.6 ± 4.1	100.8 ± 4.2	100.1 ± 3.7	100.3 ± 3.9	100.4 ± 3.9	102.7 ± 4.6	100.5 ± 4.0	101.1 ± 4.4
FPG (mg/dl)	95.3 ± 10.2	96.2 ± 10.9	96.0 ± 10.6	96.1 ± 10.8	95.2 ± 10.0	96.0 ± 10.6	92.3 ± 9.4	92.8 ± 9.7
TC (mg/dl)	209.5 ± 19.1	213.4 ± 22.8	207.2 ± 18.7	209.1 ± 19.0	211.6 ± 20.7	208.2 ± 18.9	199.3 ± 17.6	197.6 ± 16.3
LDL-C (mg/dl)	135.3 ± 13.1	135.7 ± 13.9	132.4 ± 12.4	133.3 ± 12.7	136.3 ± 14.7	130.8 ± 12.1	125.3 ± 10.8	123.6 ± 9.7
HDL-C (mg/dl)	41.5 ± 4.9	43.8 ± 5.9	41.2 ± 4.7	43.3 ± 5.7	41.9 ± 5.1	43.5 ± 5.4	43.4 ± 5.8	43.1 ± 5.3
Tg (mg/dl)	163.4 ± 24.1	169.7 ± 32.8	168.2 ± 30.1	162.6 ± 22.4	167.2 ± 29.3	169.5 ± 31.7	152.8 ± 24.9*^	154.6 ± 25.7 *^
Hs-CRP (mg/l)	1.2 ± 0.6	1.2 ± 0.6	1.1 ± 0.5	1.1 ± 0.5	1.4 ± 0.6	1.3 ± 0.5	1.2 ± 0.6*	1.1 ± 0.5*
ADN (μg/ml)	7.2 ± 2.0	7.0 ± 1.8	6.9 ± 1.6	7.1 ± 1.9	7.2 ± 2.0	7.1 ± 1.9	7.4 ± 2.3	7.5 ± 2.5

Data are expressed as mean ± standard deviations (SDs). *p < 0.05 vs. baseline; ^p < 0.05 vs. placebo. Abd. Cir., abdominal circumference; Waist Cir., waist circumference; Hip Cir., hip circumference; BMI, body mass index; FPG, fasting plasma glucose; TC, total cholesterol; LDL-C, low-density lipoprotein-cholesterol; HDL-C, high-density lipoprotein-cholesterol; Tg, triglycerides; AST, alanine aminotransferase; Hs-CRP, high-sensitivity C-reactive protein; ADN, adiponectin.

### Glycemic Parameter

Glycemia did not change during the study, although a slight decreasing trend was observed in patients receiving treatment with VSL#3^®^ (Table 1).

### Lipid Profile

Total cholesterol, LDL-C, and HDL-C did not modify during the 3 months of observation, while a statistically significant Tg decrease (*p* < 0.05 vs. baseline and *p* < 0.05 vs. placebo, respectively) was observed in the group of patients being treated with VSL#3^®^ (Table 1).

### Inflammation Parameter

A statistically significant Hs-CRP reduction (*p* < 0.05 vs. baseline) was obtained in patients receiving VSL#3^®^ compared with the group being treated with placebo (Table 1).

### Cytokine

Adiponectin did not change during the study, although a slight increasing trend was observed in patients receiving treatment with VSL#3^®^ (Table 1).

### Hepatic Values

Transaminase values were reduced at the end of the trial. AST and ALT were significantly decreased vs. baseline (*p* < 0.05) and vs. placebo (*p* < 0.05), respectively. Even the γ-GT was significantly reduced in VSL#3^®^ group (*p* < 0.05 vs. baseline and *p* < 0.05 vs. placebo) compared with the placebo group. Furthermore, AST/ALT ratio was significantly lower than the VSL#3^®^ group (*p* < 0.05 vs. baseline and *p* < 0.05 vs. placebo) compared with the placebo group (Table 2).

**TABLE 2:** Hepatic values during VSL#3^®^ treatment at baseline and after 3 months.

	Placebo				VSL#3			
	Baseline		3 months		Baseline		3 months	
Total number	30		30		30		30	
Sex	Males	Females	Males	Females	Males	Females	Males	Females
AST (IU/l)	51.2 ± 9.1	53.9 ± 10.3	49.7 ± 8.9	52.8 ± 9.5	53.4 ± 10.1	52.7 ± 9.4	46.5 ± 8.8*^	44.4 ± 7.6*^
ALT (IU/l)	60.1 ± 15.1	56.7 ± 14.0	57.8 ± 14.4	57.2 ± 14.1	59.2 ± 15.0	58.3 ± 14.9	48.3 ± 10.1*^	52.7 ± 11.8*^
AST/ALT	0.85 ± 0.05	0.95 ± 0.08	0.86 ± 0.05	0.92 ± 0.07	0.90 ± 0.06	0.90 ± 0.06	0.96 ± 0.05*^	0.84 ± 0.04*^
γ-GT (IU/l)	36.1 ± 7.0	36.9 ± 7.7	35.4 ± 6.8	35.1 ± 6.6	35.8 ± 6.8	35.9 ± 6.9	28.2 ± 5.9*^	29.4 ± 6.0*^
HIS	33.9 ± 2.0	34.2 ± 2.2	34.9 ± 2.4	35.3 ± 2.6	35.1 ± 2.5	35.6 ± 2.7	34.1 ± 2.1*^	34.7 ± 2.3*^

Data are expressed as mean ± SDs. *p < 0.05 vs. baseline; ^p < 0.05 vs. placebo. AST, alanine aminotransferase; AST, aspartate aminotransferase; γ-GT, gamma-glutamyltransferase; HSI, hepatic steatosis index.

**FIGURE 1:**
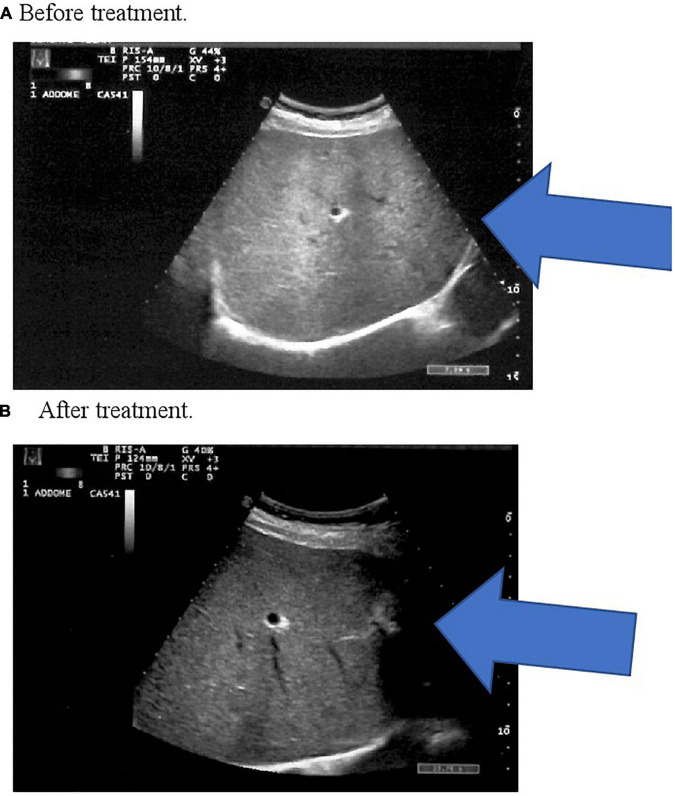
**(A)** Before treatment. **(B)** After treatment. In panel **(A)** there is an increased echogenicity of the liver, a classic sonographic finding of hepatic steatosis. In panel **(B)** ultrasound image shows normal echogenicity of the liver parenchyma.

We observed a significant decrease in HSI after 3 months of VSL#3^®^ treatment (*p* < 0.05 vs. baseline and *p* < 0.05 vs. placebo) compared with the placebo group (Table 2). All patients reported an improvement or the disappearance of hepatic steatosis (Table 3) demonstrated by the hepatic echography of patients subjected to control after 3 months of VSL#3^®^ treatment (Figure 1).

**TABLE 3:** Grade of fatty liver during VSL#3^®^ treatment at baseline and after 3 months.

	Placebo		VSL#3^®^	
	Baseline	3 months	Baseline	3 months
N	30	30	30	30
Grade 1, n (%)	16 (53.3)	14 (46.7)	19 (63.3)	5 (16.7)°**
Grade 2, n (%)	10 (33.3)	10 (33.3)	8 (26.7)	5 (16.7)*^
Grade 3, n (%)	4 (13.3)	4 (13.3)	3 (10.0)	2 (6.7)

Data are expressed as number or percentage. *p < 0.05 vs. baseline;°p < 0.01 vs. baseline; ^∧^p < 0.05 vs. placebo; **p < 0.01 vs. placebo.

### Gender Difference

No differences between men and women were registered.

## Discussion

Our study showed that all patients receiving the VSL#3^®^ probiotic had an improvement in NAFLD and also the disappearance of disease in some cases. Our data are partially different from the one reported in a previous, proof-of-concept, study (10) conducted on a little number of patients where VSL#3^®^ did not significantly improve the markers of liver injury in patients with NAFLD after 10 weeks of treatment. We think that this difference can be due to the larger number of patients enrolled, 60 vs. 35 and to the longer treatment, 12 vs. 10 weeks.

Non-alcoholic fatty liver disease is characterized by the deposition of lipids in hepatocytes and is considered the hepatic manifestation of metabolic syndrome (24). More evidence supports the thesis that dysbiosis has a role in the development of NAFLD, although very little is known about the real composition of intestinal microbiota in these patients (25). Intestinal microbes produce a vast array of bioactive molecules from any dietary compound reaching the colon, establishing an intense microbiota-host metabolism with an important impact on physiology and nutritional state (26).

Some trials observed a strong relationship between liver and gut: the portal system receives blood from the gut, and intestinal blood content can be involved in the induction and progression of liver damage in several chronic liver diseases (27). Alterations in intestinal microbiota seem to play an important role in the development of NAFLD and multiple molecular pathways have been postulated to explain the relationship between NAFLD and dysbiosis (28). There are very few trials in humans with VSL#3^®^ as probiotic supplementation. Some authors verified probiotic mixtures in patients with NAFLD. Mahboobi S et al. studied probiotic (*Lactobacillus casei*, *L. acidophilus*, *Lactobacillus rhamnosus*, *Lactobacillus bulgaricus*, *B. breve*, *B. longum*, and *S. thermophilus*) supplementation on TC, LDL-C, HDL-C, and Tg, after 8 weeks in sixty patients with pre-diabetes. They did not show any significant modification and observe that Tg value was increased by 17% (+ 16.2 mg/dl) (29). In our study, Tg was reduced by 8.7% (−14.7 mg/dl). Sepideh A et al. studied a probiotic (the same as Mahboobi et al.) vs. placebo during a period of 2 months. They reported a non-significant decrease of fasting plasma glucose (FPG) (−4.53 mg/dl, −4.6%) vs. baseline, but significantly lower vs. placebo (+ 2.62 mg/dl, + 2.7%) (30), and a significant decrease of interleukin-6 (−2.93 pg/ml, −10.0%) vs. baseline and placebo (−0.94 pg/ml, −3.1%), while we did not demonstrate any changes in FPG (−3.1 mg/dl, −3.2%), but a significant improvement of another inflammation parameter as Hs-CRP (−0.2 mg/l, −14.3%), and non-significant improvement in ADN (+ 0.3 μg/ml, + 4.2%), respectively, was observed. The transaminases amelioration was in line with the study of Aller R et al. They considered a probiotic (*L. bulgaricus* and *S. T.*) and verified a significant decrease of AST (−5.7 IU/L, −13.8%), and ALT (−7.3 IU/L, −10.8%); in our trial, we obtained a significant decrease of AST (−9.3 IU/L, −17%), and ALT (−6.7 IU/L, −11.7%), respectively (31). Another study by Nabavi S et al. aimed to verify if probiotic (*L. acidophilus* and *Bifidobacterium lactis*) yogurt consumption could modify the lipid profile and transaminases in seventy-two patients with NAFLD (32). At the end of the study, they observed a decrease of TC, LDL-C, AST, and ALT by −23.9 mg/dl (−12.2%), −20.3 mg/dl (−16.9%), −5.0 IU/L (−15.9%), and −6.0 IU/L (−19%), respectively. No significant changes were observed in the levels of FPG, Tg, or HDL-C in either group, without differentiating between men and women.

The changes of gut microbiota are important in NAFLD. Gut Gram-negative microbiota produce lipopolysaccharide and endotoxins and they start from the liver *via* the portal vein. The variations of the microbiota can lead to an increase the intestinal permeability and the endotoxins activate Kupffer cells in the liver and enhance the production of inflammatory markers and the propagations of inflammatory signals into the portal blood and the liver. Several factors have a role in modify clinical outcomes: bacterial activity of probiotic or dysbiosis of patients with NAFLD. We can read our results in this sense, considering VSL#3^®^ as a mixture of bacteria compared with other results experiencing less bacteria (33). Furthermore, the beneficial effects of probiotic compounds are associated with their metabolic activities (34). They can improve insulin resistance (35) and reduce hepatic lipids and serum endotoxin levels, which may be associated with the stimulation of expression of adenosine 5′-monophosphate (AMP)-activated protein kinase (AMPK) and serine/threonine kinase (AKT) proteins, and lipogenesis- or lipolysis-related proteins (36).

We observed discrepant results in literature, but above all, the results on the change of the hepatic grading, obtained in various studied cases in this trial, can lead us to move forward. However, to date, there are few studies with probiotics and even less in humans with VSL#3^®^. Therefore, the clinical efficacy of gut microbiota-targeted therapies on NAFLD still needs to be confirmed with large-scale and well-organized randomized clinical trials.

## Conclusion

Probiotic therapy with VSL#3^®^ ameliorates hepatic parameters and echography grading, while reducing Tg and the inflammatory status, without difference between men and women.

## Data Availability Statement

The original contributions presented in the study are included in the article/supplementary material, further inquiries can be directed to the corresponding author/s.

## Ethics Statement

The studies involving human participants were reviewed and approved by Ethics committee of Insubria University. The patients/participants provided their written informed consent to participate in this study.

## Author Contributions

GD and PM: design and conduction of the study, data interpretation, and manuscript writing. All authors contributed to data collection, reviewed the manuscript, and agreed with the content.

## Conflict of Interest

The authors declare that the research was conducted in the absence of any commercial or financial relationships that could be construed as a potential conflict of interest.

## Publisher’s Note

All claims expressed in this article are solely those of the authors and do not necessarily represent those of their affiliated organizations, or those of the publisher, the editors and the reviewers. Any product that may be evaluated in this article, or claim that may be made by its manufacturer, is not guaranteed or endorsed by the publisher.
